# Case of Severe Headache, Cured by Purgatives

**Published:** 1826-06

**Authors:** Thomas Richards

**Affiliations:** Surgeon.


**Art. III.**—*Case of severe Headache, cured by Purgatives.* By Thomas Richards, Surgeon.

As well-authenticated facts are more valuable to the medical practitioner than the most ingenious and exquisitely supported theories,—and as every such fact, if it tend to simplify or elucidate the practice of physic, is worthy of publication,—I have been induced to transmit to you the following case; not because there is any thing at all marvellous in the details, or any *interesting obscurity* in the result, but because it goes far to prove that diseases, apparently obstinate, will often yield to measures comparatively simple in character, and mild, though unerring, in effect. I am further induced to publish this case, as it will serve as a kind of appendix to the very interesting paper communicated by Dr. Granville, and inserted in your Journal for January last, in which we have seen that a very common cause gave origin to a train of symptoms exceedingly stubborn and tormenting.

Miss L— A—, aged twenty, had been labouring for some time under distressing and obstinate headache, occasionally increasing to such a degree as to render her almost frantic. To remedy her sufferings, she had recourse to leeches, with occasional doses of aperient medicine, from which she experienced only a trifling and temporary relief. As the disease seemed to gain ground upon her, she requested my attendance, and I saw her on Wednesday, the 26th of April. I found her with the following symptoms:—She complained of great pain and a sense of intolerable weight over the upper and fore part of the head, with a preponderance of both to the right side. Her eyes were considerably suffused; there was great intolerance of light and noise, with excessive languor and debility of the whole frame. The tongue was moist, and coated with mucus; and the pulse very feeble, and from fifty to fifty-four in the minute. Her feet and hands were exceedingly cold, and she had for some time been affected with a very languid circulation in the extremities; her legs and feet being covered with purple patches, with œdema of the ankles. Catamenia irregular.

As I had attended this patient before, I had reason to know that her habits were sedentary, and that the general state of her bowels was torpid: with the view, therefore, of obviating the ill effects of constipation, I had long since prescribed some aperient pills, composed of extract of colocynth and gamboge, with which I had always been led to believe she had preserved a sufficient degree of laxity of the bowels. At all events, she had taken a dose of aperient medicine that morning, which had acted freely.

Convinced, as well from my previous knowledge of her habits and constitution, as from the character of the present symptoms, that the affection of the head was not the primary disease, I felt perfectly satisfied that there was no organic injury in the brain; but, with a view to obviate any unpleasant consequences, I prescribed the application of eight leeches to the right temple, and administered a warm stomachic mixture.

The next day (Thursday,) there was no abatement of the symptoms: the headache had not been relieved by the leeches, nor had the pulse assumed a different character; the tongue was still coated, and there was the same intolerance of light and noise. Turning my attention to what I now considered the origin of all this mischief, I prescribed

℞. Hydrarg. submuriatis, gr. iij.
Extr. colocynth. c. gr. x. fiant pilulæ ij. h. s.s.

Continuatur mistura.

Friday.—The pills had produced two or three evacuations of dark, offensive scybalous matter, but the patient's sufferings were unmitigated.—Continuantur pilulæ et mistura.

Saturday.—Symptoms unaltered. Discharge from the bowels copious, dark coloured, offensive, and scybalous.—Continuantur medicamenta.

Sunday.—We now began to feel our way more distinctly. The pulse had become expanded, and risen to sixty-six; there was more warmth of the extremities, and less suffusion of the eyes; the bowels had acted very freely, and discharged the same offensive matter: still the headache was quite as urgent. I therefore repeated the application of the leeches to the temple, and prescribed—℞. Elaterii, gr. $\frac{1}{4}$.
Hydr. submuriat. gr. ij.
Pulver. jalapæ, gr. x. fiat pulvis cras mane sumendus.—The mixture to be continued as before.

Monday.—A considerable degree of excitement had been produced in the system by the opening medicine, which produced, however, several copious evacuations, of the same character as those above mentioned; but the original symptoms were not affected by its operation. The general languor and debility were very distressing, and the headache exceedingly severe. I now applied a blister to the back of the neck, and ordered—℞. Hydr. submur. Pulv. antimonialis, āā gr. iij.
Extr. colocynth. c. gr. vj. fiant pilulæ ij. h. s.s.

Tuesday.—The pills had operated freely, and, for the first time since I had seen the patient, the headache was better; the pulse was more free and regular, and the countenance more expressive of freedom from suffering than I had yet observed it. The extremities also were warmer, and altogether there was a decided amendment. I now steadily and firmly pursued the purgative plan, and found the patient's sufferings daily diminishing under its influence. So rapidly did she now improve, that, on the thirteenth day after that on which I was consulted, she had regained her accustomed spirits, and a considerable portion of her former strength; she was entirely free from pain, and sufficiently strong to leave town for the neighbourhood of Chatham, where she intends to remain for a few days.

This case, I think, is particularly worthy of the attention of the young practitioner; for it will give him confidence in a remedy, the effect of which, although not instantly obvious, was nevertheless eventually certain, and a persistance in which, in the present instance, was, at first, indicated only by the characteristic appearance of the feculæ discharged from the intestines. It will teach him, moreover, the absolute necessity, in all obscure and indefinite cases, to trust to nothing but his own ocular examination of the patient's evacuations; and it will show him, also, the truth and practical utility of Dr. Hamilton's remarks respecting the retention of feculent matter in the bowels; a fact which he will find of the utmost importance to him in his practice, and one which he should always bear in mind.

*Charlotte-street, Fitzroy-square; May 11th, 1826.*

